# Serum Adipocyte Fatty Acid-Binding Protein 4 Levels Are Independently Associated with Radioisotope Glomerular Filtration Rate in Type 2 Diabetic Patients with Early Diabetic Nephropathy

**DOI:** 10.1155/2018/4578140

**Published:** 2018-05-27

**Authors:** Xiaoqing Ni, Yunjuan Gu, Haoyong Yu, Shenqi Wang, Ying Chen, Xinlei Wang, Xinlu Yuan, Weiping Jia

**Affiliations:** ^1^Department of Geriatrics, Affiliated Hospital of Nantong University, Jiangsu, China; ^2^Department of Endocrinology and Metabolism, Affiliated Hospital of Nantong University, Jiangsu, China; ^3^Department of Endocrinology and Metabolism, Shanghai Jiao Tong University Affiliated Sixth People's Hospital, Shanghai Diabetes Institute, Shanghai Key Laboratory of Diabetes Mellitus, Shanghai Clinical Center for Diabetes, Shanghai, China

## Abstract

Serum fatty acid-binding protein 4 (FABP4) has been linked to renal dysfunction. This study evaluated the association between serum FABP4 and the radioisotope glomerular filtration rate (rGFR) in type 2 diabetic patients (T2DM) with early diabetic nephropathy. Twenty healthy controls and 172 patients with T2DM were enrolled. Serum FABP4 and renal impairment biomarkers including urinary albumin-to-creatinine ratio (UACR), serum retinal-binding protein 4 (RBP4), urinary cystatin C-to-creatinine ratio (CysC/Cr), and neutrophil gelatinase-associated lipocalin-to-creatinine ratio (NGAL/Cr) were measured. Diethylenetriaminepentaacetic acid (99mTc-DTPA) was used to test rGFR. Serum FABP4 levels were higher in T2DM patients compared with the controls. There was no significant correlation between serum FABP4 and UACR in patients with T2DM. Multivariate stepwise regression analysis showed that, in patients with T2DM, FABP4 was significantly associated with rGFR while CysC/Cr and RBP4 were significantly associated with UACR independently. But UACR had no independent association with rGFR. NGAL/Cr had no significant correlation with either rGFR or UACR. FABP4 might be an early biomarker for diabetic nephropathy if combined with UACR.

## 1. Introduction

Diabetic nephropathy (DN) is a chronic complication of diabetes, characterized by the presence of urinary albumin excretion and/or accompanied by a gradual deterioration in the glomerular filtration rate (GFR). It affects approximately 20–40% of patients with diabetes mellitus and is recognized as the leading cause of chronic kidney disease (CKD) and end-stage renal disease [1, 2]. Patients with CKD, irrespective of etiology, are at high risk for cardiovascular disease and mortality [3]. A systematic review reported that patients with type 2 diabetes mellitus (T2DM) who underwent intensive glycemic control and lipid interventions did not show improvement in clinical outcomes including all-cause mortality and death from cardiovascular causes, incident kidney failure, and nonfatal cardiovascular events [4]. Although the pathogenesis of DN remains unclear, evidence indicates that early recognition and intervention of DN may delay the progression to end-stage renal disease and cardiovascular disease [5]. However, diagnosis of DN is often delayed since the symptoms are usually insidious and develop slowly.

Considering the significant effect of CKD, Kidney Disease Outcomes Quality Initiative (KDOQI) and Kidney Disease Improving Global Outcomes (KDIGO) recommended a focal point of early identification of CKD [2, 6]. According to KDIGO recommendation, GFR (estimated GFR, eGFR) and albuminuria (urine albumin-to-creatinine ratio, UACR) are the main indicators used for initial detection and staging of acute and chronic kidney disease in adults [7, 8]. GFR is a well-validated evaluation index for kidney function and albuminuria is a marker of kidney damage [8]. GFR can be measured directly using a clearance procedure or by using equations. However, the estimating equations are relatively imprecise, with approximately 10–20% of estimates deviating by more than 30% from the measured GFR [8]. Compared with eGFR, radioisotope GFR (rGFR) using diethylenetriaminepentaacetic acid (Tc-99 m DTPA), a radio-labeled pharmaceutical agent, is a more precise measurement in clinic trials [9]. And as for UACR, it was reported to have a continuous association with the risk for progression of CKD to end-stage renal disease [10] and was recommended for the detection and staging of kidney injury [8]. But in some clinical conditions, it may remain within the normal range in early-stage DN whereas the GFR probably has already decreased [11]. It is therefore necessary to identify biomarkers that are more accurate, sensitive, and clinically attainable to reflect early renal impairment in DN. Additionally, such proposed biomarkers might be valuable for large-scale programs for early screening and prediction of the prognosis of DN.

In the past decades, several biomarkers have emerged for detection of early DN besides GFR and UACR. Among them, adipocyte fatty acid-binding protein (FABP) 4 has attracted increased attention. As a member of the calycin protein superfamily, it is a small intracellular protein expressed mainly in adipose tissue and by macrophages. Its physiological role includes fatty acid storage, transportation, metabolism, and the regulation of cell proliferation and differentiation. A high circulating FABP4 concentration was reported to correlate with body weight, glucose and lipid metabolism, and atherosclerosis and was considered an early risk factor for the progression of metabolic syndrome [12–17]. FABP4 was also reported at increased concentrations in nondiabetic as well as T2DM patients with end-stage renal disease [18, 19]. Yeung et al. reported that serum level of adipocyte FABP had a significantly inverse relationship with eGFR and was independently associated with macrovascular complications and DN staging classified by albuminuria [20].

Another adipokine, serum retinol-binding protein 4 (RBP4) is a small protein synthesized mainly in the liver and adipocytes and belongs to the lipocalin family [21]. Several studies suggest that upregulation of RBP4 correlates with obesity, insulin resistance, renal dysfunction, and cardiovascular disease in patients with T2DM [21–24]. Murata et al. reported that, in 149 T2DM patients, eGFR was an independent determinant for increased serum RBP4 levels [25].

Neutrophil gelatinase-associated lipocalin (NGAL), which is produced by epithelial cells and neutrophils, has recently gained increased attention as a sensitive and specific biomarker of tubular damage [26, 27]. Nielsen et al. investigated 177 patients with T2DM and found that after a 5-year follow-up, a high level of baseline urinary NGAL was negatively associated with GFR and positively associated with the progression to macroalbuminuria [28]. Another study reported that, in T2DM patients of short duration, urinary NGAL levels may be more sensitive than UACR for monitoring DN in the early stages [29]. A recent study reported that, in T2DM patients, both serum RBP4 and NGAL concentrations significantly and positively correlated with UACR and negatively correlated with eGFR [30].

Cystatin C (CysC), a cysteine proteinase inhibitor produced by most nucleated cells [31], has been considered as a replacement for serum creatinine or even as an alternative endogenous marker for GFR [32, 33]. The plasma concentration of CysC is stable, since it can be freely filtered through the glomerular membrane and reabsorbed and catabolized by renal tubular cells [34–36]. A large number of studies have reported that plasma levels of CysC, or eGFR formulas based on CysC, were good markers of early renal dysfunction in patients with diabetes [37–39]. However, recent studies also report that using urinary CysC or the CysC-to-creatinine ratio (CysC/Cr) to estimate renal impairment is less convincing [40, 41].

Focused on rGFR as the reference in the early stages of diabetic nephropathy, the aim of the current study was to evaluate the association among renal impairment biomarkers, with rGFR, and UACR in patients with T2DM to identity a sensitive predictor for early DN.

## 2. Patients and Methods

### 2.1. Study Population

From June 2010 to January 2013, a total of 172 T2DM inpatients were enrolled at the Department of Endocrinology and Metabolism at Shanghai Jiao Tong University Affiliated Sixth People's Hospital. T2DM patients were diagnosed according to World Health Organization diagnostic criteria (1999) [42]. Patients with type 1 diabetes mellitus or secondary diabetes, malignancy, chronic liver disorders, chronic or acute inflammation, morbid obesity (body mass index (BMI) ≥ 40 kg/m^2^), familial hypercholesterolemia, or rGFR < 30 mL/min/1.73 m^2^ were excluded. Urine and serum samples were collected on the second morning of hospitalization after a 10-h overnight fast. Twenty healthy controls were selected from the epidemiological survey database of the Shanghai Caoyang community who were matched for race, ethnicity, age, gender, and BMI with the T2DM patients in this study.

The study was approved by the Shanghai Jiao Tong University Affiliated Sixth People's Hospital Ethics Committee and was conducted in accordance with the principles contained within the Declaration of Helsinki. Each patient provided written informed consent.

Information on sex, age, anthropometric parameters including height, weight, waist circumference (WC), and blood pressure was collected. BMI was calculated as weight (kg) divided by the square of height (m^2^). Venous blood samples were collected by venipuncture into vacuum tubes. Serum samples were separated within 30 min after blood sample collection, centrifuged at 3500 ×g for 10 min, and stored at −20°C.

### 2.2. Laboratory Measurements

Fasting plasma glucose, 2-h postprandial plasma glucose, total cholesterol, triglycerides, high-density lipoprotein cholesterol, and low-density lipoprotein cholesterol were tested by enzymatic procedures using an autoanalyzer (Hitachi 7600-020; Hitachi, Tokyo, Japan). Glycosylated hemoglobin A1c values were measured by high-performance liquid chromatography (Bio-Rad Laboratories, Hercules, CA). Serum RBP4 was measured with a radioimmunoassay kit (Phoenix, Belmont, CA). Serum high-sensitivity C-reactive protein (hsCRP) was measured using a particle-enhanced immunoturbidimetric assay (Dade Behring Inc., Newark, NJ). Serum FABP4, CysC, and NGAL were measured using a sandwich enzyme-linked immunosorbent assay (BioVendor Laboratory Medicine, Modrice, Czech Republic). Urinary albumin and creatinine levels were measured using the first morning void urine samples by immunonephelometry and a BN II analyzer (Siemens Diagnostics). UACR was calculated by dividing the urinary albumin concentration by the urine creatinine concentration. Urine CysC/Cr ratio and NGAL-to-creatinine ratio (NGAL/Cr) were also calculated. The rGFR was directly measured by 99mTc-DTPA.

Diabetic patients were divided into three groups according to UACR level [7]: normal albuminuria group (UACR < 30 *μ*g/mg); microalbuminuria group (30 *μ*g/mg ≤ UACR < 300 *μ*g/mg); and macroalbuminuria group (UACR ≥ 300 *μ*g/mg). According to the rGFR stratum, T2DM patients were also divided into three subgroups [7]: normal renal function (rGFR ≥ 90 mL/min/1.73 m^2^); mild renal dysfunction (60 mL/min/1.73 m^2^ ≤ rGFR < 90 mL/min/1.73 m^2^); and moderate renal dysfunction (30 mL/min/1.73 m^2^ ≤ rGFR < 60 mL/min/1.73 m^2^).

### 2.3. Statistical Analysis

Continuous variables with a normal distribution are shown as mean ± standard deviation (SD). Data that did not have a normal distribution determined using the Kolmogorov-Smirnov test were logarithmically transformed before analysis and are shown as median (interquartile range). If data were still not normally distributed after transformation, they were analyzed using the Wilcoxon rank sum test. One-way ANOVA or the *χ*^2^ test was used for comparisons between groups, and correlations between variables were adjusted using partial correlation. Stepwise multivariate regression analysis was used to determine correlation of variables with rGFR or UACR as dependent variables. All analyses were performed using SPSS statistical package version 18.0 (SPSS, Chicago, IL, USA). A *P* value < 0.05 was considered statistically significant.

## 3. Results

As shown in Table 1, there were no significant differences between T2DM patients and healthy controls in terms of age, smoking status, BMI, lipid profiles, RBP4, rGFR, hsCRP, and CysC/Cr. Patients with T2DM had a significantly larger waist circumference, higher blood pressure, HbA1c, fasting plasma glucose, 2-h plasma glucose, UACR, FABP4, and NGAL/Cr compared with the control group (all *P* < 0.05). When compared with the control group separately, the macroalbuminuria subgroup had significantly higher levels of RBP4 and hsCRP (both *P* < 0.05), and microalbuminuria and macroalbuminuria subgroups had significantly lower rGFR (both *P* < 0.05). Among the three diabetic subgroups, patients with microalbuminuria were relatively older (*P* = 0.034). In the macroalbuminuria subgroup, the number of patients using angiotensin II receptor blockers (ARB)/angiotensin-converting enzyme inhibitors (ACEI) was significantly higher (*P* = 0.046), and systolic blood pressure (SBP), total cholesterol, triglycerides, low-density lipoprotein cholesterol, RBP4, hsCRP, and UACR were significantly higher than the other two subgroups (all *P* < 0.05). Among the three diabetic subgroups, rGFR levels decreased with increasing UACR (both *P* < 0.0001). FABP4, NGAL/Cr, and CysC/Cr were not significantly different among the three subgroups.

Based on rGFR stratum, among the three diabetic subgroups, age, diabetes duration, percentage of hypertension, WC, SBP, RBP4, UACR, and FABP4 all increased with the decrease in rGFR (all *P* < 0.05) (Table 2). For NGAL/Cr, although there were no significant differences among the three subgroups, when compared with the control group, NGAL/Cr increased in the two subgroups with rGFR less than 90 mL/min/1.73 m^2^ (both *P* < 0.05). CysC/Cr showed no significant differences between the control and diabetic group or among the three diabetic subgroups.

Correlation analysis showed that Lg FABP4 significantly correlated with age, BMI, SBP, WC, and rGFR (all *P* < 0.05, Table 3). After adjustment for covariates including sex, age, BMI, SBP, and WC, Lg FABP4 was independently associated with HbA1c (*P* = 0.022) and rGFR (*P* < 0.0001).

Multivariate stepwise linear regression analysis showed that UACR was associated with CysC/Cr and RBP4, while rGFR was associated with FABP4 and UACR (all *P* < 0.0001, Table 4). After adjustment for sex, age, BMI, SBP, WC, HbA1c, and use of medications such as ARB and ACEI, only FABP4 was significantly associated with rGFR (*P* < 0.0001), and UACR remained significantly associated with CysC/Cr and RBP4.

## 4. Discussion

In the present study, among individuals with different renal function status, we found that FABP4 levels were significantly higher in T2DM patients compared with healthy controls. Elevated FABP4 levels were independently associated with the reduction in rGFR in T2DM patients irrespective of UACR levels. CysC/Cr and RBP4 significantly correlated with UACR.

Previous studies have reported that increased FABP4 levels were associated with the deterioration in renal function in both humans and animals and therefore served as an independent indicator for the progression of nephropathy [20, 43, 44]. The mechanisms behind the elevation of FABP4 in patients with diabetic kidney disease are not yet fully understood. It is known that FABP4 is abundantly expressed in adipocytes, macrophages, and endothelial cells [45–47]. Firstly, it is suggested that, during the early stage of DN, accumulation of active macrophages is more evident in the kidney because of the elevation in oxidative stress and chronic inflammation, which consequently induce increased expression of serum FABP4 [12, 20]. Secondly, damage to glomeruli and tubulointerstitium might result in both decreased glomerular filtration and increased tubular reabsorption, leading to an increase in FABP4 in the circulation [44]. Thirdly, Okazaki et al. firstly reported that urinary excretion of FABP4 was associated with progression of proteinuria and renal dysfunction in healthy subjects [48]. The authors suggested that U-FABP4 reflects damage of glomerular with the hypothesis proposed by Tanaka et al. that main source of U-FABP4 is derived from ectopic expression of glomerular FABP4 rather than increased adiposity and that locally increased FABP4 in the glomerulus affects renal dysfunction [49]. However, there is no report about the relationship of U-FABP4 and renal dysfunction in patients with T2DM up to date.

Although UACR is the most widely used indicator for diabetic kidney damage and has been shown to predict the progression of chronic renal disease in patients with diabetes even with normal eGFR [50–52], there was a negative correlation between rGFR and UACR in the current study. Similar to our results, Cabré et al. also reported that FABP4, but not UACR, was independently associated with eGFR in T2DM patients with eGFR ≥ 60 mL/min/1.73 m^2^ [44]. However, it is unlikely that a single biomarker predicts the impairment of DN since the pathophysiological processes during the course of the disease are complex. Therefore, serum FABP4 along with UACR or a panel of biomarkers might be more sensitive for the detection of early DN.

In the pathogenesis and progression of DN, both glomerular dysfunction and tubulointerstitial damage play crucial roles. As a tubular damage marker, either in plasma or in urine, NGAL is reported to increase in the early stage of DN and can be a predictor for kidney disease progression independent of GFR [28, 30]. Results of previous studies are not conclusive, as Nauta et al. reported that urine NGAL was independently associated with UACR irrespective of eGFR [53], while Chou et al. reported that urine NGAL may not be a predictive factor associated with a decline in GFR in patients with T2DM [54]. In the current study, we found that urine NGAL/Cr did not have a significant correlation with either UACR or rGFR. It is thought that the tubulointerstitial injury in our T2DM patients with early renal impairment may be less severe, which might be the reason for the negative correlation between NGAL/Cr and UACR or rGFR. We also found that UACR significantly correlated with CysC/Cr and RBP4. This finding is consistent with previous studies which reported that both the two biomarkers were sensitive for the detection of DN in the early stage [55–57].

A strength of the current study is that rGFR was measured using the radio isotopic method, which allowed for the reliable and accurate measurement of renal function. We used urinary creatinine to correct NGAL and CysC to reduce biological variability [58].

This study had some limitations. First, the study was a single-center cross-sectional study with a small number of patients. Secondly, to confirm the role of FABP4 in the prediction of renal impairment in T2DM, a follow-up data regarding the change of rGFR in the higher FABP4 group and lower FABP4 group would be needed. However, the present study was only a cross-sectional study lacking a completed follow-up data. Thirdly, as a hospital-based study, most patients with T2DM enrolled in the study might have other macro- or microvascular complications, which may potentially affect the level of FABP4. Therefore, larger scale and multicenter prospective studies with long-term follow-up periods are required to validate the results.

## 5. Conclusion

Serum FABP4 had an inverse correlation with rGFR and could be an independent predictor for early DN. Greater reliability and a more sensitive analysis might be achieved by evaluating early diabetic renal function using UACR and a panel of biomarkers such as RBP4 and CysC/Cr in combination. Further prospective studies with long-term follow-up periods are needed to confirm the results.

## Figures and Tables

**Table 1:** Characteristics of the T2DM patients and controls according to albuminuria stratums.

	Control	T2DM	T2DM			*P*^‡^	*P*^*∗*^
			Normoalbuminuria UACR < 30 *μ*g/mg	Microalbuminuria 30 *μ*g/mg ≤ UACR < 300 *μ*g/mg	Macroalbuminuria UACR ≥ 300 *μ*g/mg		
*n*	20	172	91	52	29	-	-
Male/female	7/13	103/69	50/41	36/16^#^	17/12	0.033	0.242
Age (years)	58.0 ± 12.3	59.3 ± 13.6	56.9 ± 14.2	63.4 ± 11.7	58.4 ± 15.6	0.675	0.034
Diabetes duration (years)	-	10.1 ± 7.9	9.2 ± 7.8	11.7 ± 8.8	9.8 ± 6.0	-	0.263
Current smokers, *n* (%)	2 (10)	29 (16.9)	16 (17.5)	9 (17.3)	4 (13.8)	0.430	0.889
Hypertension, *n* (%)	-	84 (48.8)	38 (41.3)	30 (57.7)	16 (55.2)	-	0.141
ARB/ACER use, *n* (%)	-	36 (20.9)	13 (14.1)	13 (25.0)	10 (34.5)	-	0.046
BMI (kg/m^2^)	23.8 ± 4.5	23.9 ± 3.5	24.0 ± 3.6	23.4 ± 3.4	24.7 ± 3.4	0.836	0.251
Waist (cm)	81.0 ± 13.0	88.8 ± 11.4	88.9 ± 11.3^#^	88.2 ± 10.7^#^	89.8 ± 13.2^#^	0.006	0.834
SBP (mmHg)	115.0 (20.0)	130 (20)	129.0 (20.0)^†^	137.5 (25.0)^†^	140.0 (30.0)^†^	<0.0001	0.010
DBP (mmHg)	75.0 (10.0)	80 (10)	80.0 (10.0)^#^	80.0 (14.0)^#^	80.0 (10.0)	0.015	0.489
HbA1c (%),	5.6 (0.7)	8.4 (3.5)	8.1 (3.3)^†^	9.0 (3.6)^†^	9.0 (3.5)^†^	<0.0001	0.763
FPG (mmol/L)	5.1 (0.6)	7,4 (3.9)	7.2 (4.1)^†^	7.6 (4.9)^†^	7.2 (2.8)^†^	<0.0001	0.787
2 h PPG (mmol/L)	6.0 (1.8)	12.6 (7.5)	12.4 (7.3)^†^	12.9 (7.9)^†^	12.6 (7.0)^†^	<0.0001	0.897
TC (mmol/L)	5.2 (0.9)	4.9 (1.6)	5.0 (1.5)	4.4 (1.5)	5.8 (1.7)	0.803	<0.0001
TG (mmol/L)	1.4 (1.1)	1.4 (1.1)	1.4 (1.0)	1.2 (0.7)	2.3 (1.9)	0.411	0.001
HDL (mmol/L)	1.4 (0.6)	1.2 (0.5)	1.2 (0.5)	1.1 (0.5)	1.1 (0.3)^#^	0.387	0.280
LDL (mmol/L)	3.0 ± 1.0	3.0 (1.1)	3.0 ± 1.2	2.6 ± 0.8^#^	3.4 ± 1.1	0.784	0.003
RBP4 (mg/L)	41.5 (15.0)	45.0 (15.0)	43.0 (16.0)	44.0 (11.0)	58.0 (21.0)^†^	0.149	<0.0001
rGFR (mL/min/1.73 m^2^)	104.9 ± 27.9	92.2 ± 27.8	101.0 ± 25.1	85.1 ± 23.2^#^	77.0 ± 33.4^#^	0.055	<0.0001
UACR (*μ*g/mg)	5.2 (5.4)	26.2 (125.8)	8.0 (9.9)	76.9 (91.4)^†^	527.6 (1172.7)^†^	<0.0001	<0.0001
hsCRP (mg/L)	0.9 (0.1)	1.2 (3.0)	1.0 (0.3)	1.3 (0.2)	1.8 (0.4)^#^	0.257	0.014
FABP4 (*μ*g/L)	1.4 (0.9)	2.6 (4.3)	2.9 (4.0)^†^	1.9 (4.6)^†^	4.5 (6.4)^†^	0.003	0.293
NGAL/Cr (*μ*g/mg)	0.2 (1.4)	0.9 (2.1)	0.9 (2.1)^#^	0.8 (2.5)^#^	1.4 (4.9)^#^	0.018	0.219
Cys-C/Cr (*μ*g/mg)	1.7 (2.3)	2.5 (2.1)	2.5 (2.1)	3.4 (3.8)	4.6 (5.8)	0.304	0.064

Data are means ± standard deviations (SD), *n *(%) or median (interquartile range).* P*^‡^ for comparisons of diabetic patients versus control subjects; *P*^*∗*^for comparisonsamong the three diabetic subgroups;^#^*P *< 0.05 and ^†^*P *< 0.001 for comparisons between diabetic subgroups versus control subjects (by the independent *t-*test for normally distributed data or the Mann–Whitney *U *test for non-normally distributed data). UACR, urinary albumin- to-creatinine ratio; ARB, angiotensin II receptor blockers; ACEI, angiotensin-converting enzyme inhibitors; BMI, body mass index; WC, waist circumference; SBP: systolic blood pressure; DBP, diastolic blood pressure; FPG, fasting plasma glucose; 2 hPPG, 2 h postprandial plasma glucose; TC, total cholesterol; TG, triglycerides; HDL, high-density lipoprotein; LDL, low-density lipoprotein; RBP4, retinal-binding protein 4; rGFR, radioisotope glomerular filtration rate; hsCRP, high-sensitivity C-reactive protein; FABP4, fatty acid-binding protein 4; NGAL/Cr, neutrophil gelatinase associated lipocalin-to-creatinine ratio; Cys-C/Cr, cystatin C-to-creatinine ratio.

**Table 2:** Characteristics of the T2DM patients and controls according to the rGFR stratum.

	Controls	T2DM			*P*^*∗*^
		rGFR ≥ 90 mL/min/1.73 m^2^	60 ≤ rGFR < 90 mL/min/1.73 m^2^	30 ≤ rGFR < 60 mL/min/1.73 m^2^	
*n*	20	96	51	25	-
Male/female	7/13	59/37^#^	31/20	13/12	0.683
Age (years)	58.0 ± 12.3	54.0 ± 13.2	64.7 ± 8.4^#^	68.9 ± 14.9^#^	<0.0001
Diabetes duration (years)	-	8.1 ± 6.5	10.9 ± 8.1	16.5 ± 9.6	<0.0001
Current smokers (%)	2 (10.0)	18 (18.8)	7 (13.7)	4 (16.0)	0.735
Hypertension, *n* (%)	-	31 (32.2)	33 (64.7)	20 (80.0)	<0.0001
ARB/ACER use, *n* (%)	-	16 (16.7)	15 (29.4)	5 (20.0)	0.194
BMI (kg/m^2^)	23.8 ± 4.5	23.5 ± 3.5	24.8 ± 3.9	23.9 ± 2.7	0.121
WC (cm)	81.0 ± 13.0	86.8 ± 10.6^#^	92.3 ± 11.8^#^	89.8 ± 12.2^#^	0.017
SBP (mmHg)	115.0 (20.0)	123.5 (20.0)^ #^	137.5 (20.0)^ †^	140.0 (20.0)^ †^	<0.0001
DBP (mmHg)	75.0 (10.0)	80.0 (10.0)^ #^	80.0 (15.0)^ #^	80.0 (20.0)	0.746
HbA1c (%)	5.6 (0.7)	9.0 (3.5)^ †^	8.0 (2.9)^ †^	7.9 (3.3)^ †^	0.129
FPG (mmol/L)	5.1 (0.6)	7.4 (4.9)^ †^	6.9 (2.9)^ †^	7.8 (1.9)^ †^	0.372
2 h PPG (mmol/L)	6.0 (1.8)	11.7 (7.9)^ †^	13.4 (8.1)^ †^	12.6 (5.8)^ †^	0.611
TC (mmol/L)	5.2 (0.9)	5.1 (1.4)	4.7 (1.8)	4.7 (2.1)	0.302
TG (mmol/L)	1.4 (1.1)	1.3 (1.1)	1.7 (1.3)	1.3 (1.7)	0.150
HDL (mmol/L)	1.4 (0.6)	1.2 (0.5)^ #^	1.1 (0.4)^ #^	1.2 (0.5)^ #^	0.878
LDL (mmol/L)	3.0 ± 1.0	3.1 (1.1)	2.8 (1.2)	2.8 (1.1)	0.308
RBP4 (mg/L)	41.5 (15.0)	45.2 ± 12.1^#^	46.8 ± 11.3^#^	52.5 ± 12.6^#^	0.049
rGFR (mL/min/1.73 m^2^)	104.9 ± 27.9	112.2 ± 16.6	76.7 ± 8.5^†^	47.4 ± 9.8^†^	<0.0001
UACR (*μ*g/mg)	5.2 (5.4)	17.1 (62.0)^ #^	38.9 (118.6)^ †^	262.3 (473.6)^ †^	<0.0001
hsCRP (mg/L)	0.9 (0.1)	1.0 (0.3)	1.5 (0.2)	1.7 (0.5)	0.468
FABP4 (*μ*g/L)	1.4 (0.9)	2.1 (2.6)^ #^	2.6 (8.0)^ †^	5.0 (9.2)^ †^	<0.0001
NGAL/Cr (*μ*g/mg)	0.2 (1.4)	0.6 (2.1)	0.9 (1.9)^ #^	1.7 (5.1)^ #^	0.095
Cys-C/Cr (*μ*g/mg)	1.7 (2.3)	2.8 (2.9)	3.2 (2.2)	6.2 (8.4)	0.339

Data are means ± SD, *n *(%), or median (interquartile range). *P*^*∗*^for comparisonsamong the three diabetic subgroups; ^#^*P *< 0.05 and ^†^*P *< 0.001 for comparisons between diabetic subgroups versus control subjects (by the independent *t *test for normally distributed data or the Mann–Whitney *U *test for non-normally distributed data). rGFR, radioisotope glomerular filtration rate; ARB, angiotensin II receptor blockers; ACEI, angiotensin-converting enzyme inhibitors; BMI, body mass index; WC, waist circumference; SBP: systolic blood pressure; DBP, diastolic blood pressure; FPG, fasting plasma glucose; 2 hPPG, 2 h postprandial plasma glucose; TC, total cholesterol; TG, triglycerides; HDL, high-density lipoprotein; LDL, low-density lipoprotein; RBP4, retinal-binding protein 4; UACR, urinary albumin-to-creatinine ratio; hsCRP, serum high-sensitivity C- reactive protein; FABP4, fatty acid-binding protein 4; NGAL/Cr, neutrophil gelatinase associated lipocalin-to-creatinine ratio; Cys-C/Cr, cystatin C-to-creatinine ratio.

**Table 3:** Correlation analysis for Lg FABP in T2DM patients.

	Lg FABP4^†^			
	*r*	*P*	*r* ^§^	*P* ^§^
Sex^*∗*^	0.286	<0.0001		
Age (years)	0.195	0.015		
Diabetes duration (years)	0.095	0.249		
BMI (kg/m^2^)	0.272	0.001		
WC (cm)	0.292	<0.0001		
SBP^*∗*^ (mmHg)	0.190	0.023		
HbA1c (%)^†^	0.068	0.427	0.205	0.022
TC^*∗*^ (mmol/L)	0.004	0.961	−0.138	0.133
TG^*∗*^ (mmol/L)	0.140	0.086	0.081	0.355
HDL^*∗*^ (mmol/L)	0.092	0.260	0.132	0.129
LDL (mmol/L)	−0.028	0.734	0.019	0.830
RBP4 (mg/L)	0.082	0.337	0.001	0.987
rGFR (mL/min/1.73 m^2^)	−0.350	<0.0001	−0.349	<0.0001
hsCRP (mg/L)^†^	0.148	0.083	0.166	0.162
UACR^*∗*^ (*μ*g/mg)	0.057	0.481	0.067	0.435
Cys-C/Cr (*μ*g/mg)^†^	0.009	0.915	0.042	0.633
NGAL/Cr (*μ*g/mg)^†^	0.134	0.097	0.082	0.340

^†^log-transformed variables; Pearson correlation analysis for normally distributed variables; ^*∗*^Spearman correlation analysis for non-normally distributed variables; ^§^adjustment for sex, age, BMI, WC, and SBP used partial correlation analysis. FABP4, fatty acid-binding protein 4; BMI, body mass index; WC, waist circumference; SBP: systolic blood pressure; TC, total cholesterol; TG, triglycerides; HDL, high-density lipoprotein; LDL, low-density lipoprotein; RBP4, retinal-binding protein 4; rGFR, radioisotope glomerular filtration rate; hsCRP, high-sensitivity C-reactive protein; UACR, urinary albumin-to-creatinine ratio; Cys-C/Cr, cystatin C-to-creatinine ratio; NGAL/Cr, neutrophil gelatinase associated lipocalin-to-creatinine ratio.

**Table 4:** Multivariable regression analysis of various biomarkers versus UACR or rGFR.

	UACR		rGFR	
	Standard *β*	*P*	Standard *β*	*P*
Model 1				
FABP4	0.124	0.109	−0.364	<0.0001
NGAL/Cr	0.110	0.137	−0.200	0.006
CysC/Cr	0.307	<0.0001	−0.091	0.235
RBP4	0.440	<0.0001	−0.163	0.034
UACR			−0.250	<0.0001
Model 2				
FABP4	0.107	0.289	−0.349	<0.0001
NGAL/Cr	0.065	0.455	−0.095	0.220
CysC/Cr	0.295	0.001	−0.077	0.331
RBP4	0.441	<0.0001	−0.159	0.045
UACR			−0.232	0.001
Model 3				
FABP4	0.063	0.488	−0.367	<0.0001
NGAL/Cr	0.082	0.343	−0.052	0.554
CysC/Cr	0.450	<0.0001	−0.046	0.652
RBP4	0.519	<0.0001	−0.114	0.250
UACR			−0.118	0.235

Model 1: crude model without covariate adjustment; model 2: adjustment for age, sex, BMI, waist circumference, SBP, HbA1c, and use of ACEI/ARBs; model 3: adjustment for age, sex, BMI, WC, SBP, use of ACEI/ARBs, and all the other potential damage biomarkers in the table. UACR, urinary albumin-to-creatinine ratio; rGFR, radioisotope glomerular filtration rate; FABP4, fatty acid-binding protein 4; NGAL/Cr, neutrophil gelatinase associated lipocalin-to-creatinine ratio; Cys-C/Cr, cystatin C-to-creatinine ratio; RBP4, retinal-binding protein 4.
